# Engaging a Community Council for Latino Dementia Research: Findings from a Mixed-Methods Study

**DOI:** 10.1093/geroni/igaf122.1485

**Published:** 2025-12-31

**Authors:** Sara Masoud, Janna Lesser, Juana Escareno, Belinda Flores, Byeong Yeob Choi, Carole White, Karla Daniela López-Lorenzo

**Affiliations:** UT Health San Antonio, San Antonio, Texas, United States; UT Health San Antonio, San Antonio, Texas, United States; UT Health San Antonio, San Antonio, Texas, United States; UT Health San Antonio, San Antonio, Texas, United States; UT Health San Antonio, San Antonio, Texas, United States; UT Health San Antonio, San Antonio, Texas, United States; Texas A&M University, College Station, Texas, United States

## Abstract

Latino communities are disproportionately affected by Alzheimer’s disease, yet they remain underrepresented in dementia research. To address this issue in alignment with community engaged frameworks, we formed a Steering Council (SC) (N = 16) of caregivers, individuals living with dementia, community health workers, health and social service providers, and researchers. The SC worked with the study team to design and implement six “community pláticas” among different South Texas communities. These gatherings were carefully designed to integrate culturally-specific components (e.g., music, food, language translation), with a key component being reciprocal group discussions aimed at identifying research priorities and patient-centered outcomes. To assess whether strategies to engage the SC were effective, an explanatory sequential mixed-methods design study was employed. The Research Engagement Survey Tool (REST) was administered at three time points, revealing statistically significant increases in quality of engagement over time (p < 0.05 across multiple engagement principles) and strengthened levels of engagement (collaboration and partnership). Additionally, 10 in-depth interviews with SC members provided insight into the dynamics of engagement, emphasizing the evolution of trust, co-learning, and collective leadership over time. Integrated analysis confirmed that iterative, community-driven engagement strategies contribute to stronger, more sustainable research partnerships. This study offers a replicable framework for inclusive research engagement. Findings reinforce the need for culturally-aligned, patient- and family-centered research models that reflect the needs, perspectives, and experiences of populations typically excluded from research.

